# COVID-19 Vaccination Acceptance among Health Science Students in Morocco: A Cross-Sectional Study

**DOI:** 10.3390/vaccines9121451

**Published:** 2021-12-08

**Authors:** Mohamed Khalis, Mouna Boucham, Amy Luo, Abdelghafour Marfak, Soukaina Saad, Camara Mariama Aboubacar, Soukaina Ait El Haj, Manar Jallal, Fatima-Zahra Aazi, Hafida Charaka, Chakib Nejjari

**Affiliations:** 1 International School of Public Health, Mohammed VI University of Health Sciences, Casablanca 82403, Morocco; mboucham@um6ss.ma (M.B.); abmaca13@gmail.com (C.M.A.); soukai1999@gmail.com (S.A.E.H.); mjallal@um6ss.ma (M.J.); cnejjari@um6ss.ma (C.N.); 2 Harvard T.H. Chan School of Public Health, Boston, MA 02115, USA; amyluo@hsph.harvard.edu; 3 National School of Public Health of Rabat, Ministry of Health, Rabat 10000, Morocco; ab.marfak@gmail.com; 4 Laboratory of Health Sciences and Technology, Higher Institute of Health Sciences, Hassan 1st University of Settat, Settat 26000, Morocco; 5 School of Medicine, Mohammed VI University of Health Sciences, Casablanca 82403, Morocco; saadsoukaina00@gmail.com; 6 Faculty of Legal, Economic and Social Sciences Ain Chock, Hassan II University of Casablanca, Casablanca 20470, Morocco; aazi.zf@gmail.com; 7 Department of Research and Development, Ibn Sina University Hospital, Rabat 10000, Morocco; hafida572010@gmail.com

**Keywords:** COVID-19 vaccine acceptance, decision making, risk perception, public health, Morocco, Middle East and North Africa

## Abstract

While students in the health sciences occupy pivotal roles in the Moroccan COVID-19 response and vaccination campaigns, factors associated with COVID-19 vaccine acceptability among students have not been reported. This study aimed to determine the willingness and identify predictive attitudes and beliefs of COVID-19 vaccine acceptance among health science students in Morocco. A cross-sectional, self-administered online questionnaire was conducted among students of the Mohammed VI University of Health Sciences in Casablanca, Morocco in January 2021. In total, 1272 students participated. Univariate and multivariate logistic regression models were used to calculate odds ratios and 95% confidence intervals. Overall, 26.9% of participants reported being willing to receive the COVID-19 vaccine. Between genders, male students were more likely to accept the vaccine. Regarding individual attitudes and beliefs about COVID-19 infection, students with greater confidence in COVID-19 information, and higher perceived likelihood and perceived severity of infection were more likely to be willing to get the vaccine. Concerning a COVID-19 vaccine, students who reported lower levels of perceived harm and higher levels of perceived vaccine effectiveness were more willing to get vaccinated. Our findings help guide future efforts to tailor communication and identify strategies to increase COVID-19 vaccine uptake among students.

## 1. Introduction

In January 2020, the outbreak of coronavirus disease 2019 (COVID-19) was declared a global health emergency of international concern by the World Health Organization (WHO) [1]. At the time of this study, COVID-19 remains a significant challenge to health systems globally, with resounding psychological, social, and economic consequences [2,3]. As of 1 December 2021, the number of confirmed COVID-19 cases has exceeded 260 billion and 5.2 million deaths globally [4]. In Morocco, the total number of confirmed COVID-19 cases has exceeded 950,000 cases for a cumulative incidence rate of 2.6% and more than 14,775 deaths since the first reported case on 2 March 2020 [5].

Population vaccination—in combination with measures of good hygiene practices and indoor ventilation, physical distancing, travel restrictions, early detection, and contact tracing—against COVID-19 infection remains one of the best strategies for pandemic control. At present, eight vaccines have been authorized by the World Health Organization for use worldwide [6,7]. Despite significant progress in this front, rising cases of COVID-19 Variants of Concern (e.g., Delta and Omicron) in multiple countries, vaccine supply shortages particularly in low- and middle-income countries, and challenges to public confidence and acceptance of vaccines jeopardize pandemic control efforts [8,9].

In light of this, the WHO has identified vaccine hesitancy as a leading global health threat [10]. In Morocco, students in the health sciences play a central role in supporting public vaccination campaigns as reliable and trusted sources of COVID-19 vaccine information. Previous studies examining acceptance in the general population indicate that vaccination decisions are influenced by the knowledge and attitudes of health care workers [11,12]. As of 29 January 2021, the rollout of Morocco’s vaccination campaign has provided vaccines free of charge to all Moroccan citizens. However, achieving pandemic control requires a concerted effort to increase vaccination uptake, with healthcare workers playing a central role in encouraging COVID-19 vaccination. Depending on biological and socio-behavioral factors, the threshold to reach herd immunity and stop transmission ranges from 55% to 82% in the population [13]. Given global urgency, promoting positive and accurate attitudes and beliefs among health workers pertaining to COVID-19 infection and vaccines is a promising tool in building public confidence and acceptance [14].

Studies examining the acceptance of the COVID-19 vaccine among healthcare providers, students in health-related fields, and the general public showed that demographic factors of age, gender, and education were significantly associated with intention to use the vaccine [12]. In addition, individual perceptions regarding perceived infection risk, infection severity, and perceived vaccine risks and benefits are suggested predictors of vaccine acceptance and hesitancy [11,15,16,17,18]. To our knowledge, this is the first study to report the vaccine acceptance rate and identify attitude and belief factors that underpin vaccination intentions among health science students in Morocco prior to the start of the national COVID-19 vaccination campaign. Specifically, this study aimed to assess predictors of vaccine acceptance using constructs of the Health Belief Model [19]. Findings illuminate potential challenges in vaccine uptake to incorporate in the planning and implementation of Morocco’s national vaccination campaign.

## 2. Materials and Methods

### 2.1. Study Design and Setting

A cross-sectional, self-administered online survey was conducted among students of the Mohammed VI University of Health Sciences in Casablanca, Morocco, in January 2021. All matriculated students of the Mohammed VI University of Health Sciences were invited to participate in an anonymous survey. The survey was distributed to all students by school email and WhatsApp, and was completed online, via Google forms, after informed consent.

### 2.2. Study Population

In total, 1272 survey responses were received. The survey was open to all 3500 matriculated students of the Mohammed VI University of Health Sciences students at the time of the study. Responses included students from the Schools of Medicine, Dentistry, Pharmacy, Nursing and Allied Health Sciences, the International School of Public Health, and Advanced School of Biomedical Engineering. Under a hypothesized frequency of 50% in population outcome factors, the minimum effective sample size was 818 participants, applying a 95% confidence level.

### 2.3. Data Collection

The questionnaire was designed based on a review of studies and existing measures investigating attitudes and behaviors around COVID-19 vaccinations [11,12,13]. Questions were translated from English to French. Each survey question was evaluated and approved by a panel of experts from each school to test for question neutrality. Questionnaire reliability and validity was tested in a pilot study of 10 participants. Pilot participants included students from each school, and the mean age of participants were close to the mean age of the study population. Participants were asked to provide comments about questionnaire comprehension and to suggest alternatives. Results from the pilot were incorporated in the final design.

The questionnaire topics were organized in four sections adapted based on existing survey tools on vaccine hesitancy. The first section collected participant sociodemographic characteristics (age, gender, and university college affiliation), and general and COVID-19-related health information (presence of chronic disease, personal history of COVID-19 infection, having a family member, friend or classmate infected with COVID-19, loss of a loved one/or a friend due to COVID-19, and confidence in the information circulating about COVID-19). The second section included 5 questions that assessed participants’ knowledge about COVID-19 in accordance with published peer-reviewed literature and WHO reports. The third section examined the knowledge, attitudes, and beliefs of the COVID-19 infection and vaccine acceptance. Items included perceived likelihood of COVID-19 infection, perceived severity of COVID-19 infection, perceived impact of the COVID-19 pandemic, perceived harm of a COVID-19 vaccine, and perceived effectiveness of a COVID-19 vaccine. The fourth section measured participants’ vaccine hesitancy in the following question: “When a COVID-19 vaccine becomes available, will you get vaccinated?” Response options included “yes”, “no”, “not sure”. Participants were asked to indicate if items from a list of suggested factors would or would not matter in their decisions to receive a COVID-19 vaccine. Those who previously responded “no” or “not sure” to receiving the COVID-19 vaccine were asked to indicate whether each factor would or would not matter in their decisions around non-COVID-19 vaccinations.

Responses to the fourteen item questionnaire were scored accordingly: a 4-point Likert scale (0 = no confidence to 4 = high confidence) assessed confidence in circulating information about COVID-19 (range = 0–4 points), five items (range = 0–6 points) measured the accuracy of COVID-19 knowledge, two items examined perceived likelihood of COVID-19 infection (range = 2–8 points), one item assessed perceived severity of COVID-19 (range = 1–4 points), three items examined perceptions of the impact of the COVID-19 pandemic (range = 3–12 points), one item measured perceived risks of COVID-19 vaccine (range = 1–4 points), and one item rated perceived effectiveness of a COVID-19 vaccine (range = 1–4 points). Higher scores indicated greater perceived susceptibility and severity of COVID-19 infection and potential benefits of a COVID-19 vaccine.

### 2.4. Statistical Analysis

Data were analyzed using SPSS, version 20 (IBM Corp., Armonk, NY, USA). First, descriptive statistics were used to report the sociodemographic and health history characteristics of participants. Second, significant factors associated with vaccination acceptance, the crude and adjusted odds ratios (OR), and their 95% confidence intervals were estimated using logistic regression models. Finally, multivariate odds ratios were calculated to adjust for gender, loss of a family member due to COVID-19, score of confidence in the information circulating about COVID-19, score of perceived likelihood of COVID-19, score of perceived severity of COVID-19, score of perceived harm of a COVID-19 vaccine, and score of perceived effectiveness of a COVID-19 vaccine.

### 2.5. Ethical and Legal Considerations

The study protocol was conducted according to the principles of the Declaration of Helsinki. Review and approval of the study protocol were received by the Research Ethics Committee of Cheikh Khalifa Ibn Zaid University Hospital of Casablanca (CE_UM6SS/10/07/2020–14 September 2020). Recruited students were informed that participation in the study was voluntary and that answers would remain confidential. Study participants completed an anonymous informed consent form prior to beginning the questionnaire.

## 3. Results

### 3.1. Sociodemographic and General Information of Participants

A total of 1272 responses, or 36% of the 3500 students invited to participate, were received. Table 1 presents the sociodemographic and general information of participants. The mean age (±SD) of the participants who completed the study was 21.7 (±5.6) years. A total of 68.4% of participants identified as female. Top participant college affiliation was as follows: 54% from the Medical School and 14% from Nursing and Allied Health Sciences. A total of 13.2% of the participants reported having at least one chronic disease. Most of the participants (90.7%) had no history of COVID-19 infection, yet a large portion of the participants (80.4%) had family members, friends or classmates become infected with COVID-19. Finally, 25.2 % of participants had experienced the loss of a loved one or friend due to COVID-19. 

### 3.2. Attitudes toward COVID-19 and COVID-19 Vaccine Acceptance

Table 2 presents factors associated with the acceptance of the COVID-19 vaccine in this study. Overall, 26.9% of participants (343/1272) reported intentions to receive the vaccine.

In the univariate analysis (Table 2), the acceptance of the COVID-19 vaccine was found to be associated with gender (*p* < 0.01) and loss of a family member due to COVID-19 (*p* < 0.05). Participant confidence and perceived risk and benefits were associated with the likelihood of receiving the vaccine. Specifically, items measuring scores of confidence in the information circulating about COVID-19 (*p* < 0.01), perceived likelihood of COVID-19, perceived severity of COVID-19 (*p* < 0.01), perceived harm of a COVID-19 vaccine (*p* < 0.01), and perceived effectiveness of a COVID-19 vaccine (*p* < 0.01). Other factors such as age, presence of chronic disease, personnel history of COVID-19 infection, history of COVID-19 infection in family members/or friends, score of knowledge, and score of perceived pandemic impact were not found as factors predicting COVID-19 vaccine acceptance.

In multivariable analyses (Table 2), male participants were more likely to be willing to get vaccinated (AOR = 1.45, 95% CI: 1.05–2.00; *p* = 0.02)., along with participants that scored higher in the confidence in information circulating about COVID-19 (AOR = 1.43, 95% CI: 1.14–1.80, *p* < 0.01), perceived likelihood of COVID-19 (AOR = 1.23, 95% CI: 1.07–1.41, *p* < 0.01), and perceived severity of COVID-19 (AOR = 1.26, 95% CI:1.02–1.55, *p* = 0.03). Participants with lower scores regarding perceived harm of a COVID-19 vaccine (AOR = 0.32, 95% CI: 0.25–0.41, *p* < 0.01) and higher scores of perceived effectiveness of a COVID-19 vaccine (AOR = 6.1, 95% CI: 4.32–8.66, *p* < 0.01) were found more likely to be willing.

### 3.3. Factors Influencing COVID-19 Vaccination Acceptance

Potential factors underpinning decisions to receive a COVID-19 vaccine are presented in Table 3. The majority of participants (83.6%) indicated knowledge of the possible side effects of the vaccine would matter in their vaccination decisions. About 42% of participants reported that their health status and that the duration of protection provided by the vaccine are important determinants in choices to get vaccinated. However, fewer participants reported their age at vaccination (24.2%), free vaccine cost or coverage by health insurance (16.2%), opinions of their friends and family members (23.7%), and vaccine country of origin (23.7%) as factors that would affect the intention to receiving COVID-19 vaccination.

### 3.4. Factors Influencing non-COVID-19 Vaccination Acceptance

Table 4 presents factors that could potentially alter reluctant and vaccine-hesitant people to decide to receive non-COVID-19 vaccines. Among all responses collected, 73.1% of participants indicated they would be reluctant to receive a vaccine. Within this group, 51.9% of all participants indicated that vaccine messages and communications could change their decision and 21.2% of all participants indicated nothing could change their opposition to getting vaccinated. Among participants that were unsure whether they would get a COVID-19 vaccination or would refuse a COVID-19 vaccine, 48.7% reported that the advice of their doctors/or any other health professionals could change their decision about non-COVID-19 vaccines. Similarly, 48.2% indicated that WHO recommendations could change their decision about non-COVID-19 vaccination. However, fewer reported that the recommendations by the Moroccan Ministry of Health (22.6%), opinions of family members or friends (17.3%), and positive vaccination media campaigns and messages (6.2%) would alter decisions about receiving non-COVID-19 vaccines. A total of 71% of responses indicated that factors, including those not included, could alter prior reluctancy to getting vaccinated. Of this sub-group of COVID-19 vaccine-hesitant or rejecting participants, 29% indicated nothing would change their decisions pertaining to the decision to refuse any non-COVID-19 vaccines.

## 4. Discussion

This study presents the intentions of health science students to receive a future COVID-19 vaccine. Demographic, health-related, attitudes, and beliefs predictors for these intentions are based on constructs of the Health Belief Model [19]. The proportion of participants that would be willing to get a COVID-19 vaccine was 26.9%. Of the 73.1% of participants that were opposed to COVID-19 vaccination, 51.9% of all participants indicated they could be influenced by vaccine recommendations and messages. A remaining 21.2% indicated no factors could change their opposition to receiving a vaccine. The factors examined influencing vaccine acceptability included individual-level confidence in the information circulating about COVID-19, perceived susceptibility to COVID-19 infection, perceived severity of COVID-19 infection, perceived harm of a COVID-19 vaccine, and perceived COVID-19 vaccine effectiveness.

The low vaccine acceptance rate of 26.9% is consistent with previous findings in Morocco among non-health-science university students of 35.3% [20]. However, is less than half the vaccine acceptance rate (68.6%) reported in survey of households following the first reported case in Morocco [21]. In comparison to vaccine acceptance rates for health science students worldwide, the vaccine acceptance rate in Morocco (26.9%) ranks the lowest among all other countries reported. Moreover, previous studies suggest significant variation in vaccine acceptance rates between and within countries, 34.9% in Egypt [22], 35% in three European countries [23], 37.3% in Uganda [24], 56% in USA [25], 58.8% in China [26], 77% in USA [27], 84.3% in China [28], 89.4% in India [29], and 94.6% in Poland [30]. However, between country income groups, a study of 6639 dental students from 22 countries found participants from low- and middle-income countries had significantly lower rates of COVID-19 vaccine acceptance compared to peers from high-income countries [31]. These findings are in line with results from a scoping review of vaccine acceptance that showed a higher tendency toward vaccine acceptance in Europe and North America [25].

Differences in vaccine acceptance were noted between gender, with male students, on average, being more likely to receive the vaccine. In the Health Belief Model, demographic factors, such as gender and age, are modifiers that affect the thoughts and attitudes in social contexts [12,19]. While this may be due to an array of complex individual and structural factors. In a systematic review of predictors of immunization, a gender-based analysis of factors influencing vaccination status indicated that gender driven barriers may be in part due to differences in trust in health care and government institutions, and the perceived risk and potential long-term negative side effects of vaccines concerning future childbearing, familial obligations, and social roles [32]. Similarly, women often considered themselves responsible for the consequences of their familial and parenting decisions which, in turn, decreased their confidence in the use of immunization services [32]. Of note, among studies examining vaccine acceptance, gender and other demographic variables were the most inconsistent predictors of acceptance. Despite variability in the previous literature, the findings from this study support findings of significant difference in vaccine acceptance between genders due to differences.

To examine the underlying factors driving vaccine hesitancy, we assessed individual attitudes and beliefs to COVID-19. Confidence in the information and the source of information were significant predictors of vaccine acceptance. Participants who reported lower levels of confidence in the information circulating on the novel virus indicated that they were less likely to receive the vaccine. In addition, while a near majority of participants reported that vaccine recommendations from a health professional and by the WHO would likely sway those hesitant or reluctant to receive any vaccine, only a quarter of participants indicated that recommendations from the Moroccan Ministry of Health would change their decisions. These findings are in line with previous studies and indicate that the source of information (e.g., healthcare providers, government, trusted agencies, social media, trusted news sources, and communications) are important modifiers in the motivation and confidence in vaccination [12].

Further, participants more risk-averse to getting sick and with higher perceived severity of COVID-19 infection were more likely to accept the vaccine. According to the Health Belief Model, variations in vaccination intentions can be attributed to the perceived risks and benefits that guide decision-making [19,33] such that these findings align with those of previous studies; individuals who perceived themselves to be at higher risk for infection and were more concerned about the severity if they were to get sick were more willing to accept the vaccine.

The findings regarding the perceived harm of a COVID-19 vaccine indicate that those with lower perceived vaccine risk had a higher likelihood of intention to use the COVID-19 vaccine. Similar results were reported by a majority of studies conducted among health science students [22,24,25,27,34,35,36,37,38,39,40,41]. These findings suggest that concerns of serious or long-term adverse effects of a novel vaccine were important factors influencing decisions to not get vaccinated [25,27,34,36]. As reported in other contexts, concerns over the safety of the novel vaccines counterpose the perceived protective impact of the vaccine, supporting findings that vaccine compliance relies on individual risk–benefit perception [12]. Hence, education campaigns that engage health science students and provide accurate and up-to-date information on vaccine safety and effectiveness are important strategies for combatting vaccine hesitancy.

A major strength of this study is that it is the first to our knowledge to determine the vaccine acceptance rate and factors influencing decisions among university students in the health sciences in Morocco. This descriptive, cross-sectional survey included a large sample size and was representative of student from diverse health professions, including medicine, dentistry, pharmacy, nursing, public health, and biomedical engineering. In addition, the survey was carried out a few days before the start of the national vaccination campaign and is especially relevant for pandemic response and health policies.

We recognize the limitations of this study. First, the impact of vaccine hesitancy on incidence and mortality rates in this age group is unknown. While the intent to be vaccinated and general vaccine acceptance was measured before vaccines were available, what participants reported may differ from individual decisions later acted upon. Second, these findings provide an overview of acceptance among health science students. Further studies are needed to report the vaccine acceptance in the groups different from health science students, such as the general population and medical workers. Third, access to technology, internet connection, and inactivity on social media during the study period may exclude certain eligible groups. Fourth, self-reporting and social desirability biases limit the interpretation of study results as responses. Lastly, this study was conducted when information regarding COVID-19 vaccine safety and efficacy was limited in scope and before vaccines were available in Morocco, such that reported vaccine hesitancy does not delineate between attitudes between vaccine mechanisms or novelty of vaccine technology.

The findings from this study highlight the need for vaccination campaigns to address the high COVID-19 vaccine hesitancy among health science students. Unified messaging on the risks and benefits of the vaccine are key tools to encourage support for the vaccine. Specifically, these findings indicate the need to target messages for groups of health science students, such as female students, who may be more likely to refuse the vaccine. Based on these findings, further studies evaluating public health directives and effective communication strategies that address existing societal and cultural concerns are needed.

## 5. Conclusions

The findings of this study demonstrate a low acceptance rate of a COVID-19 vaccine among students in the health sciences in Morocco. Willingness to be vaccinated was associated with gender, confidence in the information circulating about COVID-19, perceived likelihood of COVID-19, perceived severity of COVID-19, perceived harm of a COVID-19 vaccine, and perceived effectiveness of a COVID-19 vaccine.

## Figures and Tables

**Table 1:** Sociodemographic and health history characteristics of participants (*n* = 1272).

Variable	Distribution *n* (%)
Gender ^1^	
Male	402 (31.6)
Female	870 (68.4)
Age (mean ± SD) ^1^	21.7 ± 5.6
≤20	652 (51.3)
>20	620 (48.7)
College	
Medicine	692 (54.4)
Dentistry	120 (9.4)
Pharmacy	73 (5.7)
Nursing and Allied Health Sciences	178 (14.0)
Public Health	123 (9.7)
Biomedical Engineering	86 (6.8)
Chronic disease	
No	1104 (86.8)
Yes	168 (13.2)
History of COVID-19	
No	1154 (90.7)
Yes	118 (9.3)
Family member, friend or classmate infected with COVID-19	
No	249 (19.6)
Yes	1023 (80.4)
Loss of a loved one, or a friend due to COVID-19	
No	951 (74.8)
Yes	321 (25.2)

^1^ Distribution is representative of the study population characteristics.

**Table 2:** Factors associated with COVID-19 vaccine acceptance (*n* = 1272).

Independent Variable	Accept (*n* = 343)	No/or Not Sure (*n* = 929)	Unadjusted		Adjusted ^1^	
	*n* (%)	*n* (%)	OR (95% CI)	*p*-Value	OR (95% CI)	*p*-Value
Gender				*p* < 0.01		
Female	183 (53.4)	687 (74.0)	Ref ^2^		Ref	
Male	160 (46.6)	242 (26.0)	2.48 (1.91–3.21)		1.45 (1.05–2.00)	0.02
Age				0.15		
≤20	187 (54.5)	465 (50.1)	Ref		Ref	
>20	156 (45.5)	464 (49.9)	0.83 (0.65–1.07)		0.76 (0.55–1.03)	0.08
Chronic disease				0.95		
No	298 (86.9)	806 (86.8)	Ref		Ref	
Yes	45 (13.1)	123 (13.2)	1.01 (0.70–1.45)		1.30 (0.83–2.04)	0.24
History of COVID-19				0.40		
No	315 (91.8)	839 (90.3)	Ref		Ref	
Yes	28 (8.2)	90 (9.7)	0.82 (0.53–1.29)		0.87 (0.52–1.48)	0.62
Family member, friend or classmate infected with COVID-19				0.10		
No	57 (16.6)	192 (20.7)	Ref		Ref	
Yes	286 (83.4)	737 (79.3)	1.30 (0.94–1.81)		1.35 (0.89–2.05)	0.15
Loss of a loved one or a friend due to COVID-19				0.05		
No	243 (70.8)	708 (76.2)	Ref		Ref	
Yes	100 (29.2)	221 (23.8)	1.31 (0.99–1.74)		1.32 (0.93–1.87)	0.11
Score of confidence in the information circulating about COVID-19	2.68 ± 0.74	2.33 ± 0.72	1.91 (1.60–2.27)	<0.01	1.43 (1.14–1.80)	<0.01
Score of knowledge about COVID-19	4.95 ± 1.01	4.86 ± 1.05	1.08 (0.96–1.22)	0.17	1.03 (0.89–1.20)	0.63
Score of perceived likelihood of COVID-19	5.33 ± 1.18	5.14 ± 1.15	1.15 (1.03–1.28)	0.01	1.23 (1.07–1.41)	<0.01
Score of perceived severity of COVID-19	3.22 ± 0.73	2.95 ± 0.78	1.59 (1.34–1.88)	<0.01	1.26 (1.02–1.55)	0.03
Score of impact of the pandemic	8.69 ± 1.69	8.79 ± 1.81	0.96 (0.90–1.03)	0.38	0.96 (0.88–1.05)	0.43
Score of perceived harm of a COVID-19 vaccine	2.31 ± 0.68	3.06 ± 0.71	0.23 (0.19–0.29)	<0.01	0.32 (0.25–0.41)	<0.01
Score of perceived effectiveness of a COVID-19 vaccine	3.30 ± 0.54	2.61 ± 0.67	8.15 (6.00–11.09)	<0.01	6.12 (4.32–8.66)	<0.01

^1^ Odds ratios adjusted for gender, loss of a family member due to COVID-19, score of confidence in the information circulating about COVID-19, score of perceived likelihood of COVID-19, score of perceived severity of COVID-19, score of perceived harm of a COVID-19 vaccine, and score of perceived effectiveness of a COVID-19 vaccine. ^2^ Reference group.

**Table 3:** Factors influencing COVID-19 vaccination acceptance (*n* = 1272).

Variable	Would Matter *n* (%)	Would Not Matter *n* (%)
Health status	538 (42.3)	734 (57.7)
Age	308 (24.2)	964 (75.8)
Free vaccine or coverage by health insurance	206 (16.2)	1066 (83.8)
Advice of your doctor	440 (34.6)	832 (65.4)
Duration of protection provided by the vaccine	540 (42.5)	732 (57.5)
Knowledge of the possible side effects of the vaccine	1064 (83.6)	208 (16.4)
Opinion of your friends or family members	302 (23.7)	970 (76.3)
Country of origin of the vaccine	301 (23.7)	971 (76.3)

**Table 4:** Factors influencing non-COVID vaccination acceptance among vaccine hesitant (*n* = 929).

Variable	Would Matter *n* (%)	Would Not Matter *n* (%)
Recommendation from a doctor or any other health professional	452 (48.7)	477 (51.3)
Moroccan Ministry of Health Recommendations	210 (22.6)	719 (77.4)
World Health Organization Recommendations	448 (48.2)	481 (51.8)
Opinion of your family members or friends	161 (17.3)	768 (82.7)
Messages conveyed in the media	58 (6.2)	871 (93.8)
Any factor, including those not included	660 (71.0)	269 (29.0)
